# Synchronous Intra-ampullary Papillary-Tubular Neoplasm and Gallbladder Cancer

**DOI:** 10.7759/cureus.105210

**Published:** 2026-03-14

**Authors:** Keita Suto, Naoya Kasahara, Masatake Taniguchi, Yoshiyuki Meguro, Yuichi Aoki, Kazue Morishima, Muhmut Amori, Kentaro Inamura, Hirotoshi Kawata, Hideki Sasanuma, Hironori Yamaguchi, Naohiro Sata

**Affiliations:** 1 Department of Surgery, Division of Gastroenterological, General and Transplant Surgery, Jichi Medical University, Shimotsuke, JPN; 2 Department of Diagnostic Pathology, Jichi Medical University, Shimotsuke, JPN; 3 Department of Clinical Oncology, Jichi Medical University, Shimotsuke, JPN

**Keywords:** ampulla of vater, gallbladder cancer, intra-ampullary papillary-tubular neoplasm (iapn), multiple tumors, pancreaticoduodenectomy

## Abstract

Intra-ampullary papillary-tubular neoplasms (IAPNs) are rare precursor lesions of the ampulla of Vater, with a high propensity for progression to invasive carcinoma. Synchronous primary malignancies of the biliary tract are uncommon, and their occurrence in the absence of congenital anomalies such as pancreaticobiliary maljunction (PBM) has not been well documented. We report a case of an IAPN coexisting with a synchronous gallbladder carcinoma in a patient without identifiable predisposing anomalies. A 70-year-old woman with a history of interstitial pneumonia, acute pancreatitis, and mixed connective tissue disease on chronic steroids was under surveillance for branch-duct intraductal papillary mucinous neoplasm of the pancreatic body. Follow-up contrast-enhanced computed tomography revealed a 14-mm enhancing mass at the ampulla of Vater, with upstream dilation of both the pancreatic and bile ducts. Endoscopic evaluation and biopsy confirmed adenocarcinoma, and she underwent pancreaticoduodenectomy. A Grade B bile leak complicated postoperative recovery, which was managed conservatively; she was discharged on postoperative day 28. Histology demonstrated a 20 × 17 mm IAPN confined to the ampullary bile duct without invasion of adjacent ducts, and immunophenotyping confirmed a gastric/pancreatobiliary phenotype. Incidentally, a separate 32 × 30 mm gallbladder fundus tumor was identified and diagnosed as primary gallbladder carcinoma invading only the muscular layer (pT1b, pN0). Resection margins were negative, and no adjuvant therapy was administered due to comorbid pulmonary disease. This case underscores the importance of thorough preoperative evaluation of the gallbladder in patients with ampullary neoplasms, even in the absence of known biliary tract anomalies. Recognition of synchronous malignancies may alter surgical planning and improve outcomes. Vigilant imaging, including targeted ultrasonography, should be incorporated into the preoperative workup for ampullary lesions to detect occult gallbladder carcinoma.

## Introduction

Intra-ampullary papillary-tubular neoplasms (IAPNs) are precancerous lesions that occur within the papilla and were defined by Ohike et al. in 2010 [1,2]. IAPNs mainly occur within the ampulla of Vater and form polypoid lesions that grow locally within the ampulla of Vater and have characteristics different from ampullary adenoma that arise in the duodenal mucosa [1]. IAPNs are considered the ampullary counterpart of intraductal neoplasms of the pancreatobiliary system, such as intraductal papillary mucinous neoplasms (IPMNs) of the pancreas and intraductal papillary neoplasms of the bile duct. The ampulla of Vater comprises three distinct epithelial components, duodenal mucosa, pancreatic ductal epithelium, and biliary epithelium, and displays a unique combination of morphological and histological features [2,3]. Diagnosing IAPNs can be challenging, particularly in small endoscopic biopsy specimens, due to their heterogeneous histology.

Pancreaticobiliary maljunction (PBM) is a congenital anomaly in which the pancreatic and bile ducts join outside the duodenal wall, leading to chronic biliary epithelial exposure to pancreatic enzymes and increasing the risk of multifocal carcinogenesis [4]. Consequently, synchronous development of gallbladder cancer and extrahepatic cholangiocarcinoma is observed in patients with PBM.

Herein, we present a rare case of synchronous primary gallbladder cancer and an IAPN in a patient without PBM. The patient underwent curative pancreaticoduodenectomy for biopsy-proven ampullary carcinoma, which revealed synchronous gallbladder cancer.

## Case presentation

A 70-year-old woman who had been admitted to our hospital with a diagnosis of branch duct IPMN in the body of the pancreas had a mass at the ampulla of Vater detected on a CT scan during follow-up. As for her medical history, she had interstitial pneumonia, acute pancreatitis, mixed connective tissue disease (MCTD), and she was taking oral steroids. She had no relevant family history. She was not a smoker and drank socially. Physical examination did not reveal any abnormalities. Her laboratory tests revealed a normal complete blood count and normal liver function. The serum carcinoembryonic antigen level was 9.3 ng/mL (normal range: <4.5 ng/mL), and the carbohydrate antigen 19-9 (CA19-9) level was <1 U/mL (normal range: <37 U/mL).

**Figure 1:**
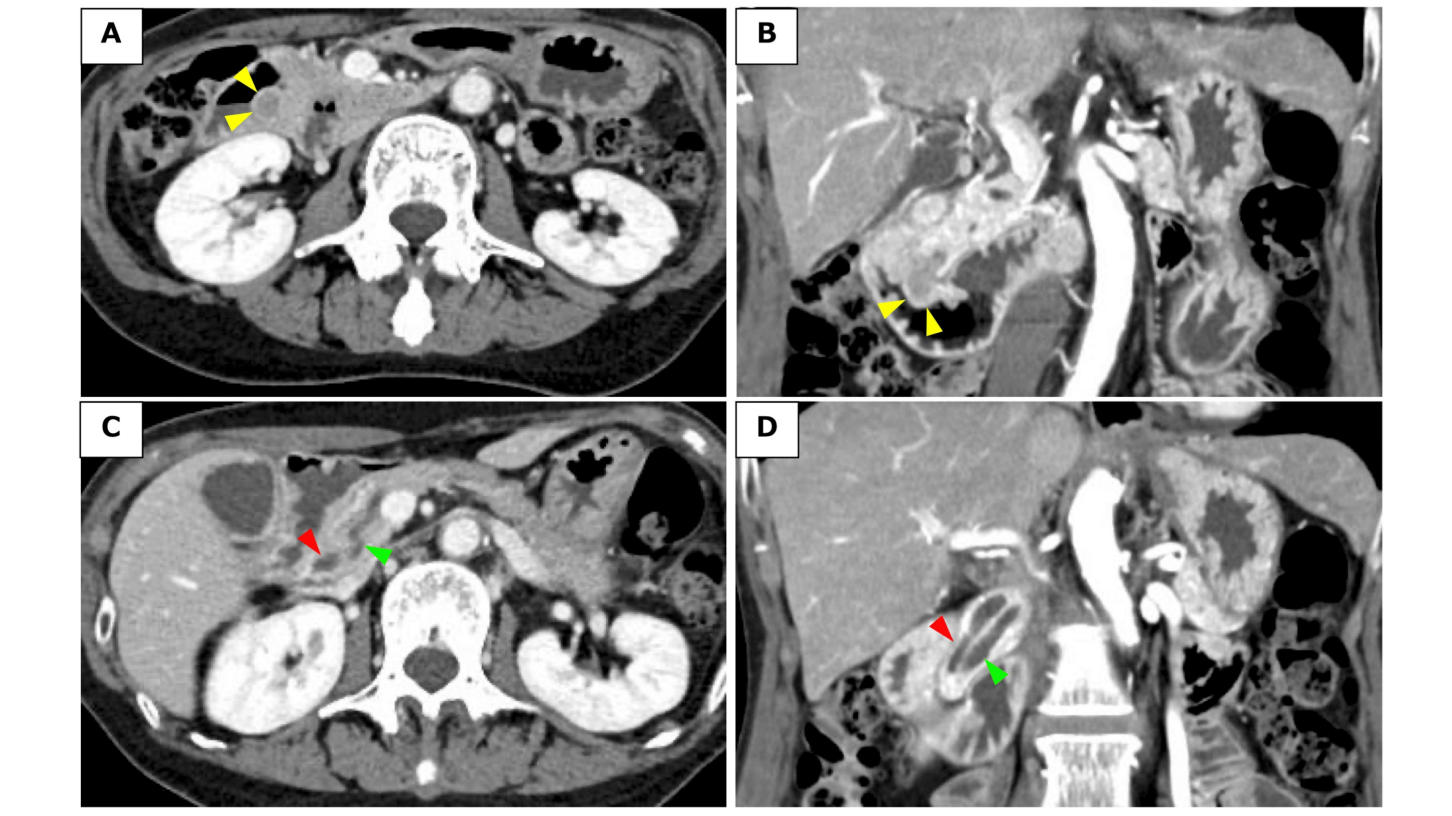
Abdominal computed tomography (CT). (A) Enhanced CT showed a 14mm mass in the ampulla of Vater (arrows) (axial image). (B) Coronal image of the tumor in the ampulla of Vater (yellow arrows). (C) CT scan revealed that the main pancreatic duct (green arrow) and common bile duct (red arrow) were dilated near the ampulla of Vater due to the tumor (axial image). (D) Coronal image of the dilated main pancreatic duct (green arrow) and common bile duct (red arrow).

Contrast-enhanced CT revealed a 14-mm arterial-enhancing mass in the ampulla of Vater (Figures 1A, 1B), along with dilation of both the main pancreatic duct and the bile duct near the ampulla (Figures 1C, 1D). Trans-abdominal ultrasonography was not performed in this case.

**Figure 2:**
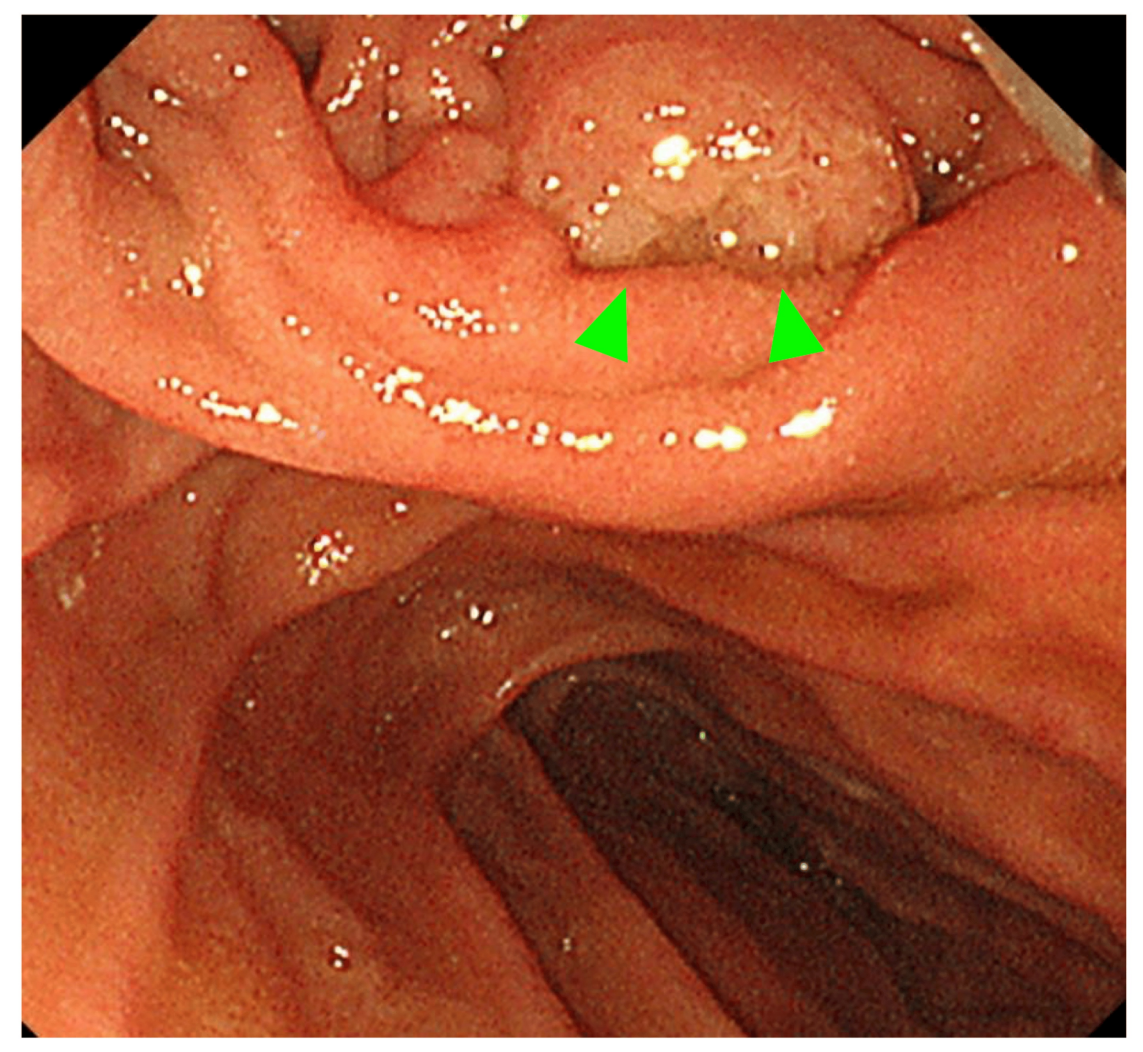
Finding on duodenoscopy. Duodenoscopy revealed a proximal bulge of the duodenal papilla, with an exposed, pale-colored mass at the orifice (arrows).

**Figure 3:**
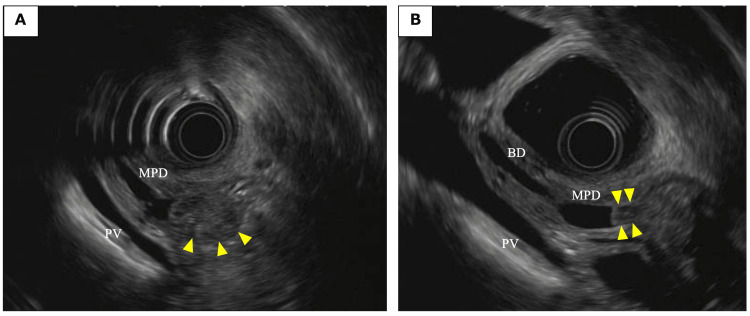
Findings of endoscopic ultrasound (EUS). (A) A hypoechoic tumor was found in the ampulla of Vater (arrows). (B) Infiltration of the main pancreatic duct (MPD) near the ampulla of Vater was suspected (arrows). BD: Bile duct; PV: portal vein

**Figure 4:**
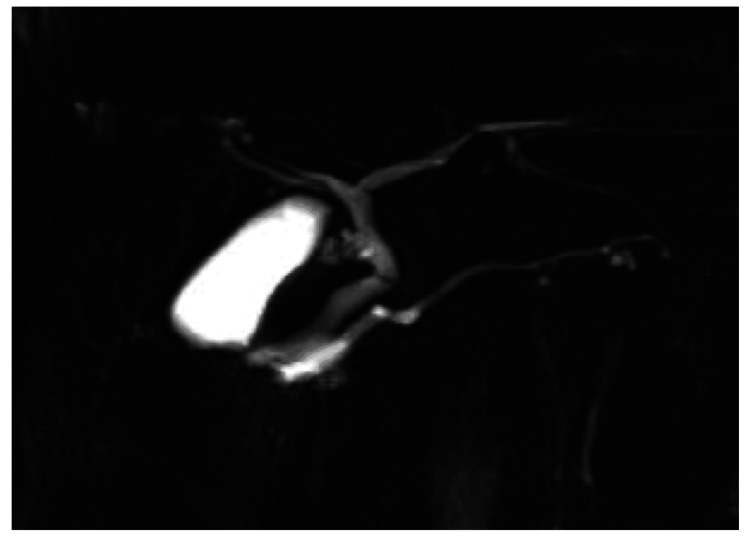
Magnetic resonance cholangiopancreatography did not reveal any pancreaticobiliary maljunction.

Duodenoscopy revealed a proximal bulge of the duodenal papilla, with an exposed, pale-colored mass at the orifice (Figure 2). Cytological and histological examination of the tumor revealed a diagnosis of adenocarcinoma. Endoscopic ultrasound (EUS) was performed, and a hypoechoic tumor was found in the ampulla of Vater (Figure 3A). The main pancreatic duct was dilated, and infiltration of the main pancreatic duct near the ampulla of Vater was suspected (Figure 3B). Magnetic resonance cholangiopancreatography was performed, and no PBM was found (Figure 4).

The preoperative diagnosis was ampullary adenocarcinoma, and the patient underwent pancreaticoduodenectomy. Postoperatively, the patient developed a bile leak classified as Grade B according to the International Study Group of Pancreatic Surgery (ISGPS) criteria [5]. Non-operative management or active intervention, percutaneous drainage, was required. The patient was discharged home on postoperative day 28.

**Figure 5:**
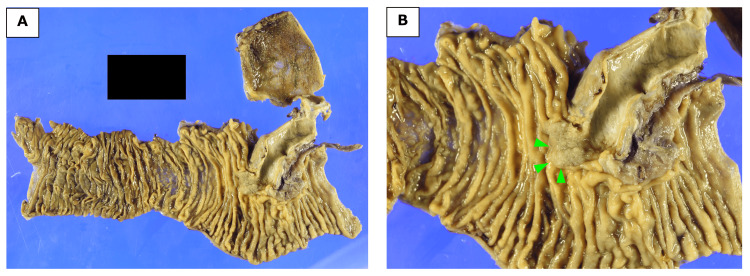
Macroscopic examination of the resected specimen. (A) Overall view of the resected specimen. (B) On macroscopic examination, a whitish tumor was located in the ampulla of Vater (arrows).

**Figure 6:**
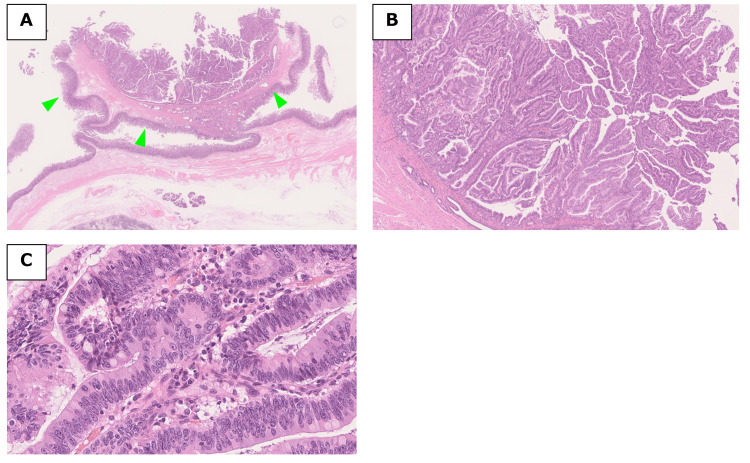
Histopathological findings of the resected specimen. (A) The tumor was localized within the common duct of the ampulla of Vater, without invasion into the pancreatic duct or common bile duct (arrows: tumor). (B) Tubulopapillary proliferation of tall columnar epithelial cells was observed in the common duct within the ampulla of Vater. (C) The tall columnar epithelial cells exhibited high-grade atypia, characterized by an increased nuclear-to-cytoplasmic (N/C) ratio.

**Figure 7:**
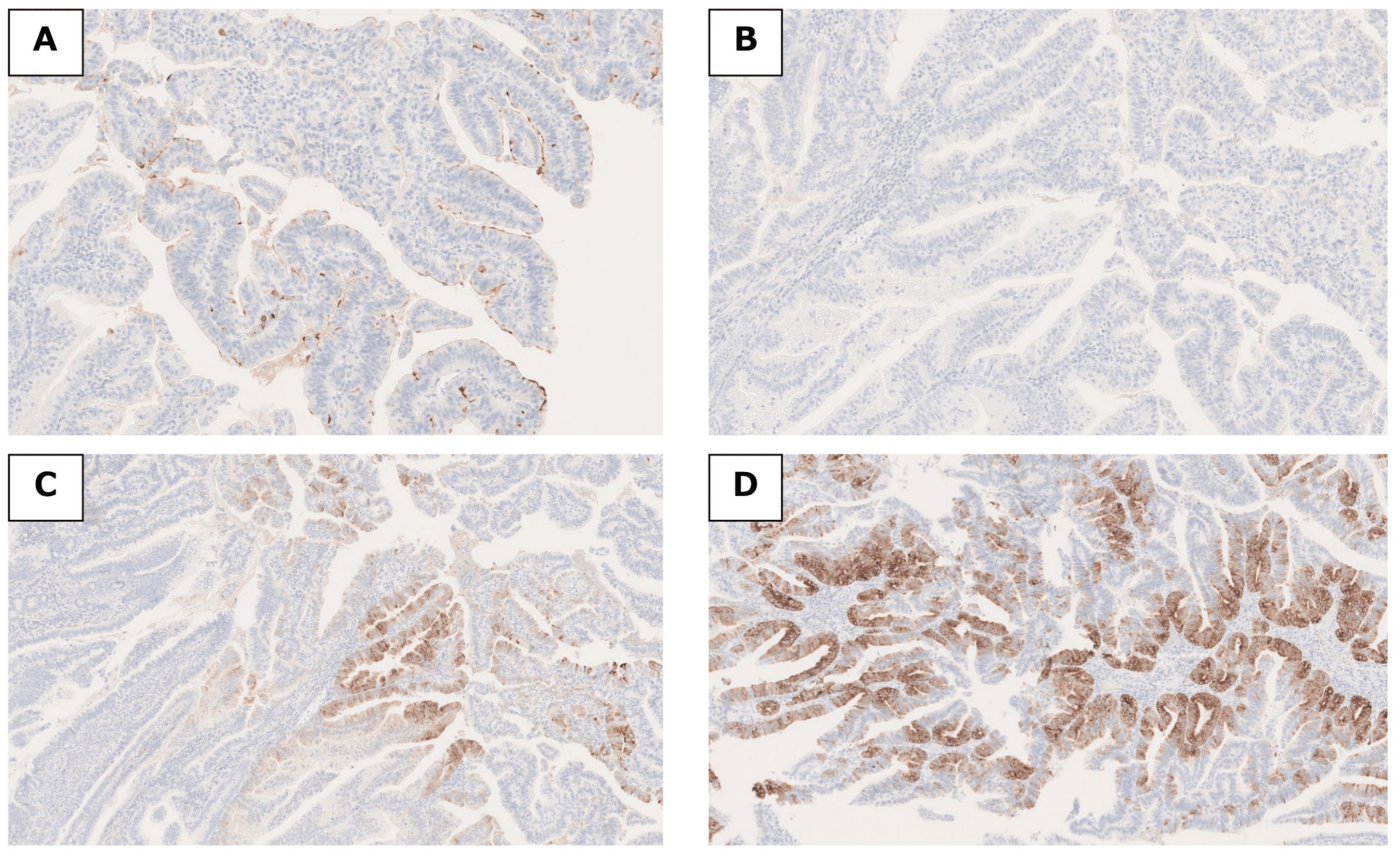
Immunohistochemical findings for the mucinous phenotype. Immunohistochemistry findings are shown for (A) mucin core protein (MUC)1,(B) MUC2, (C) MUC5AC, and (D) MUC6.

**Figure 8:**
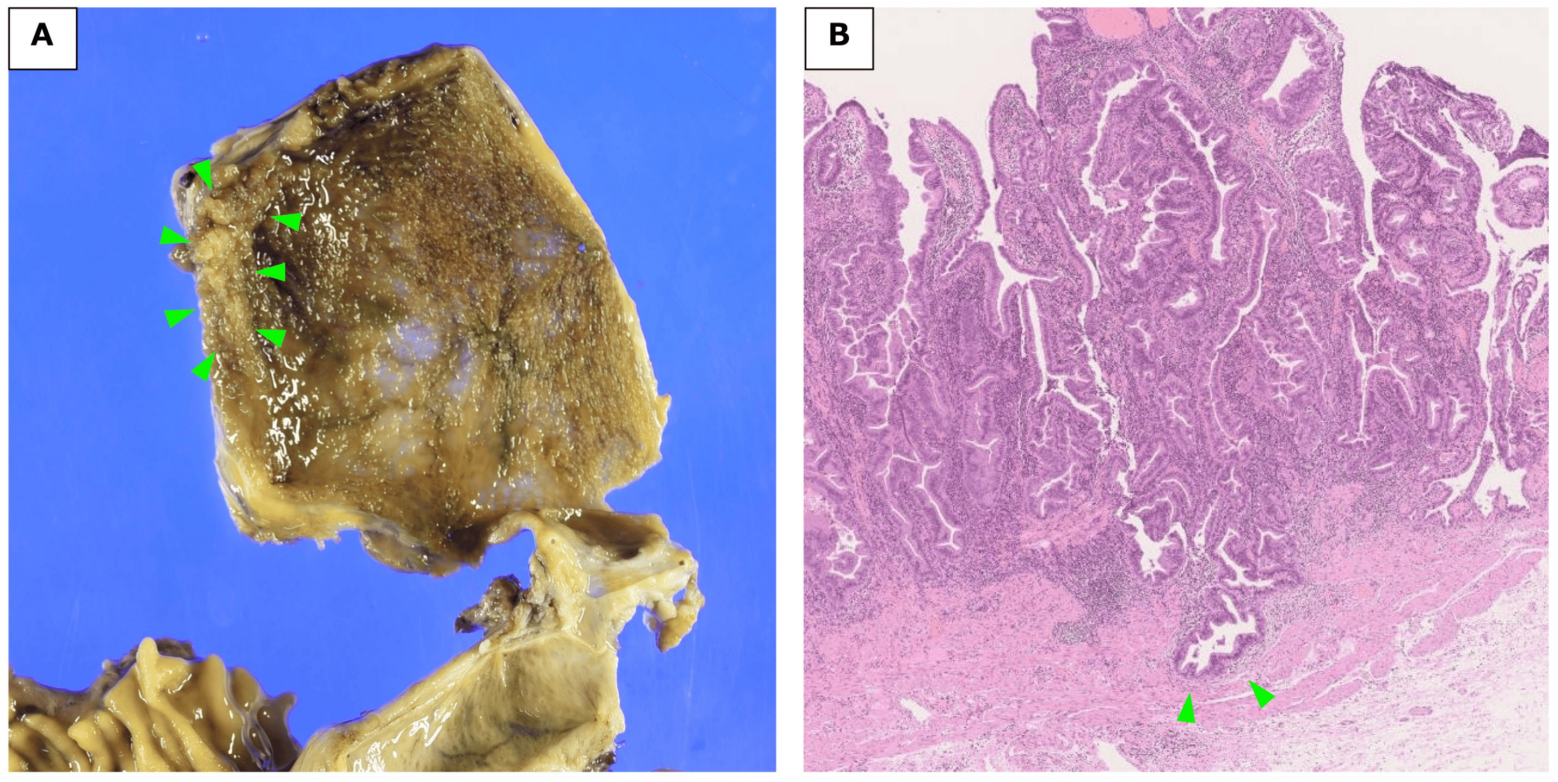
Pathological findings in gallbladder cancer. (A) The tumor measuring 32×30 mm was identified at the fundus of the gallbladder (arrows). (B) Histology revealed that the tumor invasion was limited to the muscularis propria (arrows) and did not extend to the serosa.

Histopathological examination of the resected specimen revealed a 20 × 17 mm whitish papillary mass in the ampulla of Vater (Figures 5A, 5B), without invasion into the common bile duct or main pancreatic duct (Figure 6A). The specimen also confirmed the absence of PBM. A tubulopapillary proliferation of highly atypical columnar epithelial cells with an increased nuclear-to-cytoplasmic (N/C) ratio was observed in the common duct within the ampulla of Vater (Figures 6B, 6C). The tumor was diagnosed as an IAPN. The extent of the lesion was limited to the bile duct within the head of the pancreas and had not reached the pancreatic duct. The mucosal phenotype of IAPN was positive for MUC1, MUC5AC, and MUC6, consistent with the gastric/pancreatobiliary (GPB) type (Figure 7). An incidental 32 × 30 mm tumor was identified in the gallbladder fundus and diagnosed as primary gallbladder carcinoma (Figure 8A). The tumor was completely separate from the IAPN of the duodenal papilla, with normal biliary epithelium observed between the two lesions. The tumor invaded the muscular layer but not the subserosa (pT1b) (Figure 7B), with no lymph node metastasis in the regional lymph nodes (pN0). The resection margin was negative, and the final diagnosis was pStage I.

Postoperative adjuvant therapy was not administered due to the presence of interstitial pneumonia and MCTD as comorbidities.

## Discussion

IAPNs present with a wide range of pathological findings, ranging from mild ductal epithelial atypia equivalent to hyperplasia or adenoma, to low-grade or high-grade adenocarcinoma [1,2,6]. They typically manifest as a papillary tumor within the ampulla, often causing early symptoms such as obstructive jaundice or pancreatitis, which may contribute to earlier clinical detection and relatively favorable outcomes in some cases [1-3,6,7]. However, once an invasion occurs, the prognosis worsens significantly: Reported three- and five-year survival rates are 100% for non-invasive cases, while invasive cases respectively show reduced survival rates of 69% and 45% [1,8].

The cell-lineage morphology of IAPNs is classified into intestinal (INT) and GPB types. According to Ohike et al., cell-lineage morphology of IAPNs is often INT type, whereas cases with widespread high-grade dysplasia are often GPB type, suggesting that GPB type cases are at high risk of progressing to high-grade dysplasia and invasive cancer [1]. GPB type is the most invasive, with more lymphatic and perineural invasion and a tendency toward higher malignancy. Furthermore, although the adenomatous portion may exhibit INT-type morphology, the invasive areas frequently demonstrate GPB-type carcinoma, reflecting a lack of morphological continuity [1]. This case was the GPB type, and although no invasive components were observed, it was accompanied by high-grade dysplasia.

IAPNs carry a high risk of progression to invasive carcinoma. Invasion has been reported in up to 94% of resected cases, with lymph node metastasis observed in approximately 42% of invasive cases [8]. Endoscopically, IAPNs are frequently recognized as mucosa-covered, protruding lesions that may mimic benign processes. The difficulty in preoperative assessment is particularly evident when the tumor widens the ampullary orifice without surface exposure, rendering evaluation of invasiveness highly unreliable. Notably, invasive components are frequently missed on preoperative biopsy, with only about 40% showing evidence of invasion, posing a significant diagnostic challenge [8]. As a result, lesions classified as “adenoma-like” or “high-grade adenoma” on endoscopy often prove to contain invasive carcinoma in the final pathological specimen. The ampulla of Vater is anatomically complex, and the collected specimens are small and prone to artifacts. Therefore, findings indicating true invasion, such as irregular infiltration accompanied by desmoplasia, are often difficult to confirm from biopsy specimens alone. Factors such as fragmentation, crushing, inflammatory atypia, aberrant or ectopic ducts, and tangential biopsy sectioning can lead to misdiagnosis of invasion [9]. Therefore, final pathology may not confirm suspected infiltration as seen in the present case. These findings illustrate the major diagnostic challenge of IAPNs and highlight the inherent limitations of endoscopic classification.

While EUS is useful for evaluating the extent of tumor invasion into the biliary and pancreatic ducts, its staging accuracy can be limited. One report described a 63% concordance rate between EUS findings and pathological progression [10]. EUS may overestimate or underestimate tumor invasion; therefore, although endoscopic modalities provide critical preoperative information, their findings must be interpreted within the broader clinical context.

Surgical resection is generally recommended for IAPNs when distant metastasis or unresectable invasion is absent [7]. Although endoscopic papillectomy or duodenectomy may be considered in select cases with expert endoscopic support, surgery remains the preferred approach, especially when the presence of invasion cannot be reliably excluded preoperatively. In this case, pancreaticoduodenectomy was performed based on biopsy findings suggestive of carcinoma. While no invasive component was found on final pathology, the decision for surgery was considered justified given the lesion’s high malignant potential. 

Importantly, histopathological examination revealed a synchronous gallbladder carcinoma alongside the IAPN. The two tumors were clearly separate with distinct histological characteristics and no continuity, fulfilling the criteria for true synchronous carcinoma [11]. Importantly, the two tumors were not only anatomically separated but also histopathologically independent. Normal biliary epithelium clearly intervened between the two lesions, and no transitional or continuous progression was observed. Furthermore, the ampullary tumor was a noninvasive IAPN with high-grade dysplasia, while the gallbladder lesion was an invasive adenocarcinoma confined to the muscularis, suggesting different stages and pathways of tumor development. These pathological findings strongly exclude intraductal extension or metastatic lesions and support the concept of multicentric carcinogenesis within a common carcinogenic field. 

Multiple risk factors contribute to biliary tract malignancies, including congenital anomalies like PBM and choledochal cysts, as well as inflammatory or infectious conditions such as hepatolithiasis and parasitic infections [4]. Among these, PBM is most strongly associated with synchronous cancers, observed in 62.5% of such cases [12]. However, in the present case, there was no evidence of PBM or other identifiable risk factors.

Abdominal ultrasound can non-invasively evaluate gallbladder lesions and identify findings such as wall thickening, polyps, and gallstones for risk stratification, leading to the detection of gallbladder cancer [13]. Incidental gallbladder cancer is frequently present, making it difficult to suspect malignancy preoperatively [14]. When surgery to remove the gallbladder and papillary lesion is planned as here, a thorough abdominal ultrasound as a primary screening test remains clinically significant.

An epidemiological study of cancers of the pancreas, extrahepatic bile duct, and ampulla of Vater demonstrated similar age-incidence density plots and parallel linear trends in log-transformed age-incidence curves across the four sites. These findings suggest a shared field effect in the pathogenesis of these cancers, with differences in incidence likely attributable to variations in the surface area of ductal epithelium at each site. These four tissues are derived from the same foregut. It has been reported that these cancers share a common etiology and may exhibit the so-called "field effect" regarding carcinogenesis [15]. IAPNs are described as counterparts of pancreatic IPMNs on a histologic basis. Similarly, such lesions in the gallbladder are referred to as intracholecystic papillary neoplasms (ICPNs). Although we found no pathological evidence to support an ICPN origin for the present gallbladder carcinoma, it may share a tumorigenic mechanism with IAPNs as suggested above.

In this case, despite the absence of PBM or other known predisposing factors, the two tumors were clearly separated pathologically with normal epithelium preserved between them. This finding provides clinical and histological support for the field cancerization hypothesis in the pancreaticobiliary region. In addition, this finding suggests that a common carcinogenic field may induce independent neoplastic changes at multiple sites even in the absence of congenital or inflammatory risk factors.

## Conclusions

We reported a case of a synchronous IAPN and gallbladder carcinoma occurring in the absence of PBM. Accurate preoperative assessment of invasion and malignancy in IAPNs remains challenging, as endoscopic findings and biopsy results often underestimate the extent of disease. Surgical resection remains the treatment of choice when invasion cannot be excluded. Furthermore, preoperative evaluation of the gallbladder, including ultrasonography, is essential to exclude the possibility of synchronous gallbladder carcinoma when performing pancreaticoduodenectomy for periampullary tumors.
